# Causal relationship between T2DM microvascular complications and gut microbiota: a Mendelian randomization study

**DOI:** 10.3389/fendo.2024.1349465

**Published:** 2024-06-03

**Authors:** Junping Zhang, Zilu Yu, Shanshan Li, Qingfang Zhang, Wen Chen, Jingying Wang, Shasha He, Ying Liu, Shen Chen, Jixiong Xu

**Affiliations:** ^1^ Department of Endocrine and Metabolism, The First Affiliated Hospital, Jiangxi Medical College, Nanchang University, Jiangxi, China; ^2^ Queen Mary School, Medical College, Nanchang University, Nanchang, China; ^3^ Jiangxi Clinical Research Center for Endocrine and Metabolic Disease, Nanchang, Jiangxi, China; ^4^ Jiangxi Branch of National Clinical Research Center for Metabolic Disease, Nanchang, Jiangxi, China

**Keywords:** gut microbiome, type 2 diabetes, retinopathy, neuropathy, nephropathy, Mendelian randomization study

## Abstract

**Background:**

Gowing number of studies have demonstrated the association between gut microbiome and T2DM microvascular complications, however the causal relationship remains unclear. Therefore, we using the Mendelian randomization (MR) approach to investigate this causal relation.

**Methods:**

Using gut microbiome data from the International MiBioGen Consortium genome-wide association study (GWAS) and T2DM microvascular complications data from the FinnGen Consortium GWAS to perform MR analyses. Single nucleotide polymorphisms (SNPs) were selected as instrumental variables (IVs), the inverse variance weighting (IVW) method was used as the primary analysis method, and the results were tested for heterogeneity and horizontal pleiotropy.

**Results:**

Our research identified that there are 5 known microbial species and 2 unknown microbial species in the gut microbiome that were causally related to T2DM retinopathy. Besides, three and seven known microbial species causal relationships between the gut microbiome and T2DM neuropathy and T2DM nephropathy, respectively.

**Conclusions:**

Using MR methods, we demonstrated the causal relationship between gut microbiome and microvascular complications in T2DM, providing a new strategy for the prevention and treatment of it.

## Introduction

Microvascular complications of type 2 diabetes mellitus(T2DM) refer to the complications arising from structural and functional changes in the microvasculature in T2DM. These include T2DM retinopathy, T2DM neuropathy, and T2DM nephropathy (1, 2). As the specific pathogenesis of microvascular complications in T2DM is not yet clear, and the onset of these complications is often insidious, they are typically diagnosed at a more advanced stage in clinical settings. This results in further physical and mental harm to patients with T2DM (3). Therefore, it is essential to study the causes of microvascular complications in T2DM.

Recent research has found that the gut microbiota (GM) is not only strongly correlated with T2DM but also has a certain relationship with microvascular complications of T2DM (4–6)^–^. Bai et al. discovered that, compared to the control group, the abundances of *Blautia, Collinsella, Dorea et al.* genera were reduced in T2DM retinopathy, while the abundance of *Bacteroides, Megamonas, Alistipes et al.* increased (7). The closest relationship between T2DM neuropathy and the GM lies in the enteric nervous system (ENS) changes mostly. In related animal model studies, it was found that the neural signal transmission in T2DM mice was inhibited, leading to the loss of intermuscular motor neurons, resulting in GM dysbiosis, ultimately leading to gastrointestinal neuropathy. The reduced abundance of *Allobaculum*, *Lactobacillus*, and *Bifidobacterium* might be the main reason (8). Lv et al.’s research suggested that the significant reduction of *Proteobacteria* and *Epsilonbacteraeota*, along with the increased abundance of *Bacteroides*, *Eubacterium*, and *Roseburia*, may be related to kidney damage (9). Meanwhile, Tao et al. believe that *Escherichia-Shigella* and *Prevotella*-9 might be used to distinguish T2DM patients with or without nephropathy (10). In summary, the correlations between GM and the microvascular complications of T2DM were proved. However, due to the limitations in the studied populations and methodologies, the relationships between some known or unknown microbial species and microvascular complications of T2DM remain unclear.

Mendelian randomization (MR) study is an emerging approach in recent years to study the causal relationship between diseases and exposure factors (11). Currently, a large number of studies focus solely on the correlation between GM and microvascular complications of T2DM. Considering the lack of discussion on their causal relationship, this study uses the Mendelian randomization to explore the causal relationship between microvascular complications of T2DM and the GM.

## Materials and methods

### Overview of the MR study design

**Figure 1:**
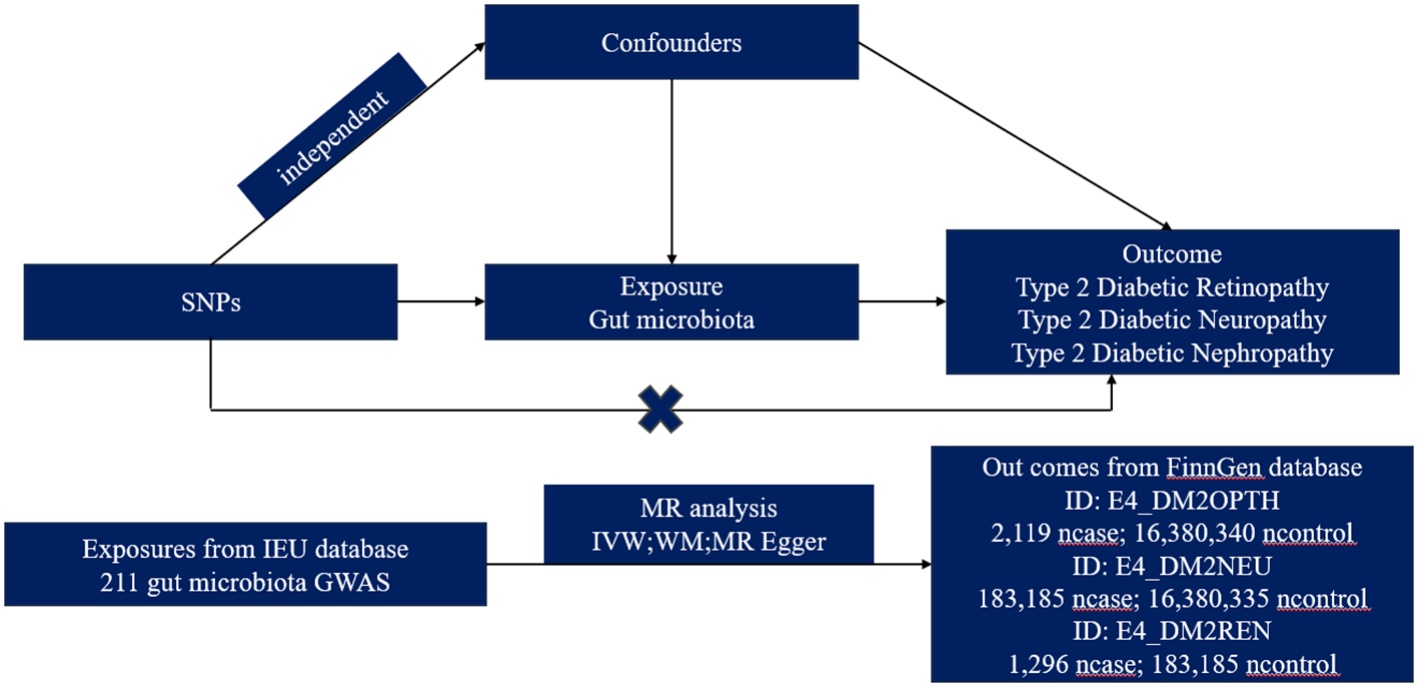
Study design for MR.

We obtained summary-level data from publicly available genome-wide relationship studies (GWAS). The GWAS study that provided data on GM was initiated by the international MiBioGen consortium (12), while the GWAS study offering data on T2DM microvascular complications was primarily sourced from the FinnGen database. The MR analysis was grounded on three pivotal preconditions: Firstly, the instrumental variables (IVs) chosen must exhibit a robust association with GM taxa. Secondly, these IVs operate independently from any confounders affecting both GM taxa and T2DM microvascular complications. Lastly, there should be no horizontal pleiotropy, meaning the IVs influence T2DM microvascular complications strictly through their effect on GM taxa (13). Ethical approval and consent to participate were secured for each cohort included in the GWAS studies, and the summary-level data were released for analysis (**Figure 1**).

### Study exposures and study outcomes

Drawing on the resources of the MiBioGen consortium, Kurilshikov et al. analyzed 16S rRNA gene sequencing profiles and genotyping data from 18,340 samples to delve into the interplay between genetic variations and GM. These samples represented subjects from 25 cohorts across 11 countries, all of European descent. From this effort, the GWAS study pinpointed 122,110 variant sites spread across 211 taxa, from genus to phylum levels, highlighting the variances in GM taxa across populations.

We utilized the R package “ieugwasr” to extract three GWAS datasets from the IEU platform (https://gwas.mrcieu.ac.uk/), their IDs are finn-b-E4_DM2OPTH, finn-b-E4_DM2NEU, and finn-b-E4_DM2REN. These datasets originate from the FinnGen research projects (https://r8.risteys.finngen.fi/phenocode/E4_DM2OPTH, https://r8.risteys.finngen.fi/phenocode/E4_DM2NEU, https://r8.risteys.finngen.fi/phenocode/E4_DM2REN). The source of the dataset is detailed in **Supplementary Table S11**.

### Statistical analysis

Statistical analyses were executed using R software (Version 4.1.1). We employed the “TwoSampleMR” R package to investigate the potential causal relationship between GM taxa and microvascular complications of T2DM. P value < 0.05 was deemed indicative of statistically significant evidence for a potential causal effect. To bolster the integrity of the data and the precision of the findings, SNPs associated with GM taxa and T2DM microvascular complications that met a genome-wide significance threshold (P < 1×10–^5^) were selected, these SNPs were further confirmed to be uncorrelated by a distance cut-off of 10,000 kilobases apart and a correlation index R^2^ ≤ 0.001. In the absence of horizontal pleiotropy, the inverse variance weighted (IVW) test was used as the primary method for calculating the causal effect values to obtain unbiased estimates. A fixed/random effects model was selected for the IVW test based on the presence or absence of heterogeneity. OR and 95% confidence interval (CI) showed the effect size. The weighted median (WM) method and the MR-Egger test were utilized as additional methods for MR analysis. WM results were used as the significant causal effect values if the number of SNPs with heterogeneity exceeded 50%. MR Egger’s results remained valid if SNPs with pleiotropy were above 50%. Cochrane’s Q test was applied to test for heterogeneity. IVs with P<0.05 were considered heterogeneous. The intercept of MR Egger regression assessed the presence of potential pleiotropy in IVs. Horizontal pleiotropy was deemed to be non-existent if P >0.05. To ensure the accuracy of results for GM taxa causally related to T2DM microvascular complications (based on IVW results), the multipotency was further analyzed using the MR-Pleiotropy RESidual Sum and Outlier (MR-PRESSO) test (R package “MRPRESSO”). And, the leave-one-out method was used to validate data robustness. Additionally, we performed a reverse Mendelian randomization analysis to further assess whether there was a causal relationship between microvascular complications of type 2 diabetes and identified significant bacteria.

## Results

All positive results related to causal relationship between the GM and T2DM microvascular complications are presented in **Table 1**. All raw results were listed in the **Supplementary Material** (**Supplementary Table S1-S9**).

**Table 1:** MR estimation of associations between GM and type 2 diabetes microvascular complications and tests for heterogeneity and horizontal pleiotropy.

GM ID in IEU	GM name	Method	Number of SNP	OR (95% CI)	P-value	Q test p-value	MR-Egger intercept test P-value	MR-PRESSO P-value
Type 2 Diabetic Retinopathy and GM								
ebi−a−GCST90016932	Family *Clostridiales vadin* BB60 group	**IVW**	**15**	**1.42(1.11–1.81)**	**0.01**	0.92	0.36	0.97
		WM	15	1.30(0.92–1.83)	0.13	-		
		MR-Egger	15	1.05(0.54–2.05)	0.89	0.92		
ebi−a−GCST90016959	genus *Actinomyces*	**IVW**	**7**	**1.43(1.16–1.92)**	**0.02**	0.98	0.86	0.98
		WM	7	1.38(0.95–2.00)	0.09	–		
		MR-Egger	7	1.34(0.65–2.78)	0.47	0.95		
ebi−a−GCST90017012	genus *Gordonibacter*	**IVW**	**12**	**0.82(0.69–0.98)**	**0.03**	0.44	0.90	0.42
		WM	12	0.87(0.68–1.12)	0.27	-		
		MR-Egger	12	0.87(0.39–1.90)	0.73	0.35		
ebi−a−GCST90017030	genus *Lactobacillus*	**IVW**	**9**	**1.33(1.05–1.68)**	**0.02**	0.99	0.65	0.82
		WM	9	1.33(0.96–1.82)	0.08	–		
		MR-Egger	9	1.16(0.62–2.16)	0.66	0.99		
ebi−a−GCST90017058	genus *Ruminococcaceae* UCG010	**IVW**	**6**	**0.58(0.39–0.88)**	**0.01**	0.74	0.32	0.22
		WM	6	0.60(0.36–1.00)	0.05	-		
		MR-Egger	6	0.31(0.10–0.97)	0.11	0.84		
Type 2 Diabetic Neuropathy and GM								
ebi−a−GCST90016949	family *Rhodospirillaceae*	**IVW**	**15**	**0.58(0.40–0.85)**	**<0.01**	0.19	0.30	0.18
		WM	15	0.60(0.36–0.99)	0.05	–		
		MR-Egger	15	1.37(0.27–6.90)	0.71	0.20		
ebi−a−GCST90016988	genus *Dialister*	**IVW**	**11**	**0.53(0.33–0.85)**	**0.01**	0.56	0.60	0.05
		WM	11	0.89(0.13–6.30)	0.06	-		
		MR-Egger	11	0.52(0.26–1.02)	0.91	0.49		
ebi−a−GCST90017072	genus *Sutterella*	**IVW**	**12**	**0.60(0.37–0.96)**	**0.03**	0.61	0.94	0.62
		WM	12	0.56(0.29–1.07)	0.08	–		
		MR-Egger	12	0.64(0.08–5.04)	0.68	0.52		
Type 2 Diabetic Nephropathy and GM								
ebi−a−GCST90016923	class *Verrucomicrobiae*	**IVW**	**11**	**1.82(1.24–2.68)**	**<0.01**	0.88	0.59	0.87
		WM	11	1.58(0.95–2.63)	0.08	-		
		MR-Egger	11	1.29(0.36–4.65)	0.71	0.85		
ebi−a−GCST90016957	family *Verrucomicrobiaceae*	**IVW**	**11**	**1.82(1.24–2.68)**	**<0.01**	0.88	0.59	0.88
		WM	11	1.58(0.97–2.57)	0.06	–		
		MR-Egger	11	1.28(0.36–4.64)	0.71	0.85		
ebi−a−GCST90016961	genus *Akkermansia*	**IVW**	**11**	**1.82(1.24–2.68)**	**<0.01**	0.88	0.59	0.87
		WM	11	1.58(0.94–2.64)	0.08	-		
		MR-Egger	11	1.28(0.36–4.63)	0.71	0.85		
ebi−a−GCST90017010	genus *Flavonifractor*	**IVW**	**5**	**1.91(1.11–3.30)**	**0.02**	0.99	0.81	0.96
		WM	5	2.00(1.00–4.00)	0.05	–		
		MR-Egger	5	1.44(0.16–12.7)	0.76	0.97		
ebi−a−GCST90017072	genus *Sutterella*	**IVW**	**12**	**0.63(0.42–0.94)**	**0.02**	0.73	0.33	0.71
		WM	12	0.64(0.37–1.10)	0.11	-		
		MR-Egger	12	0.26(0.05–1.47)	0.16	0.75		
ebi−a−GCST90017108	order *Verrucomicrobiales*	**IVW**	**11**	**1.82(1.24–2.68)**	**<0.01**	0.88	0.59	0.89
		WM	11	1.58(0.96–2.60)	0.07	–		
		MR-Egger	11	1.29(0.36–4.65)	0.71	0.85		
ebi−a−GCST90017116	phylum *Proteobacteria*	**IVW**	**12**	**0.52(0.33–0.79)**	**<0.01**	0.55	0.49	0.33
		WM	12	0.53(0.29–0.99)	0.04	-		
		MR-Egger	12	0.78(0.23–2.66)	0.70	0.50		

Bold indicates the results derived from the IVW method.

### Causal relationship between type 2 diabetic retinopathy and GM

**Figure 2:**
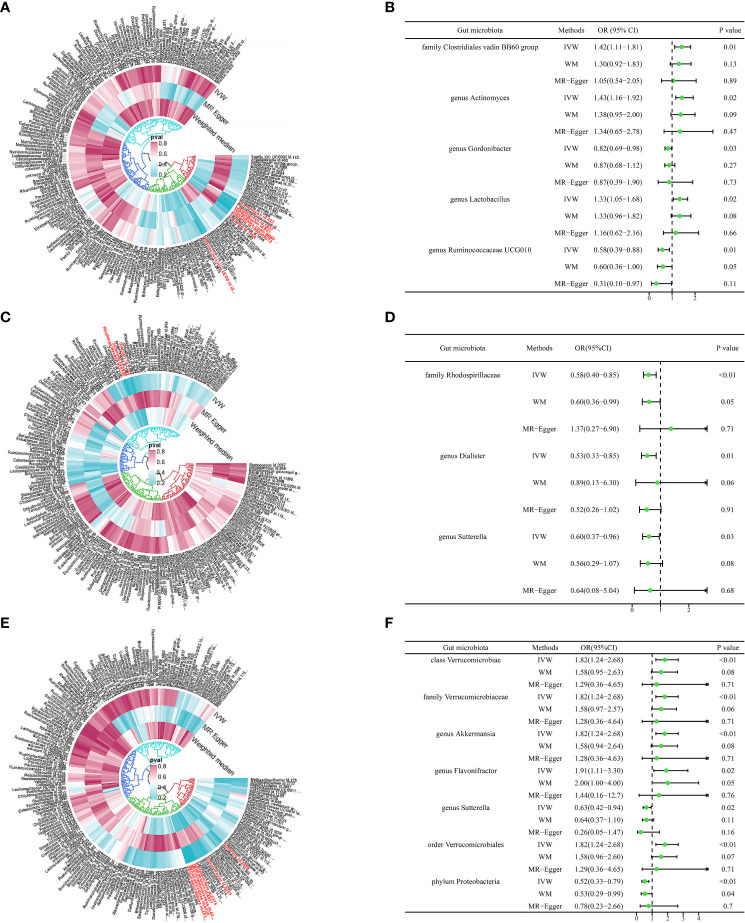
Causal analysis of gut microbiome and type 2 diabetes microvascular complications. **(A)** The microbial species causally associated with type 2 diabetic retinopathy. **(B)** MR results of microbial species with a causal relationship to type 2 diabetic retinopathy. **(C)** The microbial species causally associated with type 2 diabetic neuropathy. **(D)** MR results of microbial species with a causal relationship to type 2 diabetic neuropathy. **(E)** The microbial species causally associated with type 2 diabetic nephropathy. **(F)** MR results of microbial species with a causal relationship to type 2 diabetic nephropathy.

**Figure 3:**
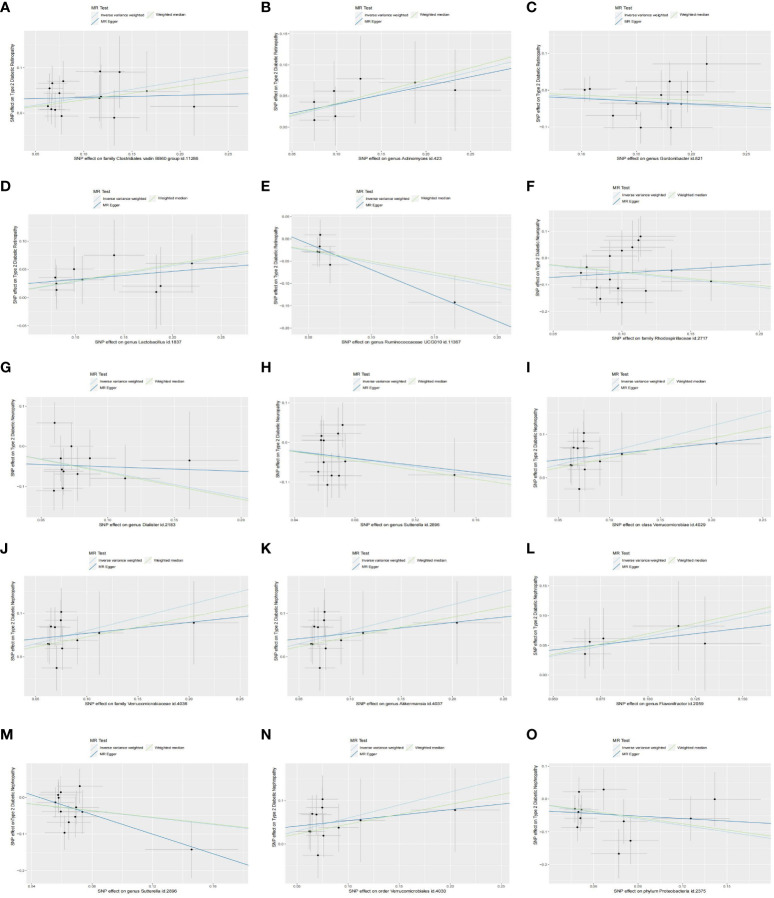
Scatter plots of the causal effect of identified bacterial taxa on type 2 diabetic microvascular complications: **(A–E)** Scatterplot of the gut microbiome causally associated with type 2 diabetic retinopathy. **(F–H)** Scatterplot of the gut microbiome causally associated with type 2 diabetic neuropathy. **(I–O)** Scatterplot of the gut microbiome causally associated with type 2 diabetic nephropathy. .

In our analysis, we employed three well-established MR methods. Utilizing the IVW approach, we pinpointed five specific microbial species with a causal association to T2DM retinopathy, as illustrated in **Figure 2A**. Notably, the Family *Clostridiales vadin* BB60 group (OR = 1.42, 95% CI: 1.11–1.81, P = 0.01), genus *Actinomyces* (OR = 1.43, 95% CI: 1.16–1.92, P = 0.02), and genus *Lactobacillus* (OR = 1.33, 95% CI: 1.05–1.68, P = 0.02) were identified as risk factors for T2DM retinopathy. Conversely, genus *Gordonibacter* (OR = 0.82, 95% CI: 0.69–0.98, P = 0.03) and genus *Ruminococcaceae* UCG010 (OR = 0.58, 95% CI: 0.39–0.88, P = 0.01) were recognized as protective factors (**Figure 2B**). Furthermore, the WM and MR-Egger methods yielded results consistent in both size and direction with those obtained through the IVW method, as depicted in **Figures 3A–E**.

### Causal relationship between type 2 diabetic neuropathy and GM

The results presented in **Table 1**, derived from the IVW analysis, provide compelling evidence suggesting that family *Rhodospirillaceae* (OR = 0.58, 95% CI: 0.40–0.85, P < 0.01), genus *Dialister* (OR = 0.53, 95% CI: 0.33–0.85, P = 0.01), and genus *Sutterella* (OR = 0.60, 95% CI: 0.37–0.96, P = 0.03) exhibit a protective effect against the development of T2DM neuropathy (**Figure 2D**). This underscores the potential significance of these microbial taxa in mitigating the risk of T2DM neuropathy. **Figure 2C** visually portrays the relationships between 211 GM and T2DM neuropathy. Notably, except for *Rhodospirillaceae*, the results from all three MR methods for the remaining species displayed similar effect sizes and orientations, as depicted in **Figures 3F–H**.

### Causal relationship between type 2 diabetic nephropathy and GM

The results depicted in **Figure 2E** reveal that seven microbial species exhibit causal associations with T2DM nephropathy. Among these, Class *Verrucomicrobiae* (OR = 1.82, 95% CI: 1.24–2.68, P <0.01), family *Verrucomicrobiaceae* (OR = 1.82, 95% CI: 1.24–2.68, P <0.01), genus *Akkermansia* (OR = 1.82, 95% CI: 1.24–2.68, P <0.01), genus *Flavonifractor* (OR = 1.91, 95% CI: 1.11–3.30, P = 0.02), and order *Verrucomicrobiales* (OR = 1.82, 95% CI: 1.24–2.68, P <0.01) were identified as risk factors for T2DM nephropathy. In contrast, the genus *Sutterella* (OR = 0.63, 95% CI: 0.42–0.94, P = 0.02) and phylum *Proteobacteria* (OR = 0.52, 95% CI: 0.33–0.79, P <0.01) were identified as protective factors against T2DM nephropathy (**Figure 2F**). The consistency of the results across the three MR methods (**Figures 3I–O**) adds further strength to these findings, indicating that these microbial species and taxonomic groups play a consistent role in either increasing or decreasing the risk of T2DM nephropathy.

### Sensitivity analysis

We use IVW and MR-Egger tests to detect heterogeneity, and the results indicate no heterogeneity among all the IVs (P <0.05, **Table 1**). In addition, the MR-PRESSO test the MR-Egger regression intercept test also showed no horizontal pleiotropy (**Table 1**). And leave-one-out analyses suggested that the MR results are robust and not influenced by specific SNPs (**Supplementary Figure S1**).

### Bidirectional MR findings

We used type 2 diabetes microvascular complications as an exposure factor and the fifteen identified significant gut microbial taxa as the outcomes for reverse MR analysis. The results showed that no significant causality was detected, except for genus *Sutterella* (OR = 1.05, 95% CI: 1.01–1.09, P = 0.02), which was reciprocally associated with type 2 diabetic nephropathy. The results of the reverse MR analysis are shown in **Supplementary Table S10**.

## Discussion

Our research employed advanced MR techniques to investigate the potential causal links between the GM and three microvascular complications of T2DM, namely retinopathy, neuropathy, and nephropathy. As a result of our analysis, we discovered a total of seven microbial species, including two unidentified ones, that exhibited associations with retinopathy. In the case of neuropathy, we found three distinct microbial species to be linked. Furthermore, our investigation revealed a connection between seven microbial species and nephropathy. The implications of these findings are noteworthy, as they hold promise for advancing our understanding of T2DM microvascular complications. This knowledge may prove valuable in the development of strategies for preventing or early detecting these complications in the future.

### Retinopathy

Our discovery unequivocally demonstrates a causal relationship between retinopathy and specific taxonomic groups within the microbial community. Notably, the Family *Clostridiales vadin* BB60 group, as well as the genera *Actinomyces*, *Gordonibacter*, *Lactobacillus*, and *Ruminococcaceae* UCG010 were found to be intricately linked to the development of retinopathy. This insight contributes significantly to our understanding of the role these microbial entities play in the occurrence and progression of retinopathy.

Research into the connection between *Clostridiales* and retinopathy has been relatively limited. In a previous clinical trial conducted by Huang et al (14), the diabetic retina group exhibited decreased levels of *Clostridium* genera and increased levels of *Lactobacillus* when compared to the normal control group. However, in a foreign study (15), it was observed that patients with both (T2DM) and metabolic syndrome had elevated levels of *Clostridiales*. Concerning *Actinomyces*, its role in GM has been a subject of debate. Some studies have depicted it as beneficial for human health (16), while others have contested this notion. Relevant animal studies have shown that a high-fat diet can significantly elevate the presence of intestinal *Actinomyces (* 17), and given that obesity was a primary contributor to the high prevalence of T2DM, which can lead to severe complications. Li et al. found that obesity can exacerbate retinopathy by increasing *Actinobacteria* levels, further supporting this view (18). The impact of *Lactobacillus* on diabetes remains unclear. As mentioned earlier, it can be increased in diabetic retinopathy patients (14). Nevertheless, in another study conducted by Li et al (19), it was found that *Lactobacillus* was reduced in the diabetic retina group compared to the diabetic group without retinopathy. *Ruminococcaceae* UCG010, belonging to the phylum Firmicutes, has shown potential in ameliorating metabolic disorders and inflammation during diabetes by influencing the production of Short-chain fatty acids (SCFAs) and the conversion of primary to secondary bile acids (BAs) (20, 21). In a MR study exploring the gut microbiome and T2DM, it was revealed that the risk of T2DM was inversely associated with *Ruminococcaceae* UCG010, consistent with our study, thus reinforcing the reliability of our findings (21). Furthermore, our research extends the understanding of the relationship between *Ruminococcaceae* UCG010 and diabetic retinopathy, providing a promising direction for future investigations in this field.

To date, no relevant studies have explored the association between *Gordonibacter* and T2DM retinopathy.

### Neuropathy

Our research also uncovered a significant causal relationship between the microbial taxa *Rhodospirillaceae*, *Dialister*, and *Sutterella* with respect to T2DM neuropathy.

Remarkably, most studies focusing on *Rhodospirillaceae* have been conducted in animal models. Pei et al. conducted an animal study in which mice were divided into a normal diet group and a high-sugar diet group (HGD) (22). After 16 weeks of feeding, the HGD group exhibited a notable increase in the abundance of *Rhodospirillaceae* in the GM, accompanied by the development of neuromuscular dyskinesia. The findings from this study slightly differ from our research, and it’s possible that the disparity can be attributed to the variation in subjects studied, i.e., animals versus humans. The role of *Dialister* in T2DM patients has been a subject of contention. A Romanian cohort study suggests that *Dialister* may potentially have deleterious effects in T2DM (23). However, a systematic review has indicated a decrease in *Dialister* abundance in patients with newly diagnosed T2DM (24, 25). On the other hand, *Sutterella*, a gram-negative bacterium, has shown associations with diabetes (26). In their study, Gaike et al. observed that, in comparison to the healthy group, the abundance of *Sutterella* was reduced in newly diagnosed T2DM patients, hinting at a potential protective role in the development of T2DM (27). Nonetheless, none of the prior studies have delved further into the relationship between these two microbial species and T2DM neuropathy. This innovative aspect of our study not only sheds new light on this specific connection but also paves the way for future research in this domain.

### Nephropathy

According to our research, we have identified a causal relationship between several microbial taxa and T2DM nephropathy, including the class *Verrucomicrobiae*, orders, families, the genera *Akkermansia*, *Flavonifractor*, and *Sutterella*, as well as the phylum *Proteobacteria*.

In a comparative analysis of the gut microbiome composition between patients with T2DM and healthy individuals, a significant increase in the abundance of *Verrucomicrobia* was observed at the phylum level (28). Additionally, research conducted in T2DM mouse models indicated a substantial increase in *Verrucomicrobia* at multiple taxonomic levels, including phylum, class, and order, implying its potential involvement in accelerating the development of T2DM (29). Salguero et al. conducted a study comparing the T2DM chronic kidney disease group with healthy controls and found that *Verrucomicrobia* exhibited increased abundance in patients within the kidney disease group (30). These findings align with the results of our study, supporting the notion that *Verrucomicrobia* may play a role in the progression of T2DM nephropathy. Surprisingly, investigations into the relationship between *Akkermansia* and nephropathy have predominantly centered around animal experiments, yet consistent results remain elusive. In the study conducted by Shi et al (31), it was discovered that Huangkui capsules could enhance the metabolic profiles of mice with diabetic nephropathy by modulating the GM, notably by decreasing the abundance of *Akkermansia*. Conversely, Luo et al. found that inulin-type fructans could lead to the enrichment of *Akkermansia*. This enrichment, in turn, ameliorated glomerular injury and renal fibrosis by elevating serum acetate concentration (32). These contrasting findings underscore the complexity of the relationship between *Akkermansia* and nephropathy, indicating the need for further research to unravel the underlying mechanisms and potential therapeutic applications in this context. Regarding *Flavonifractor*, in an experiment that involved the comparison of GM between individuals with diabetic nephropathy (DN) and those with diabetes alone, a noteworthy observation emerged. It was evident that *Flavonifractor* exhibited a marked increase in the DN group in contrast to the diabetic-alone group. Furthermore, subsequent investigations have robustly supported these findings, revealing a significant and positive correlation between *Flavonifractor* and parameters such as urinary albumin/creatinine ratio and thylakoid stromal hyperplasia (33). The consistency of these results with our own study highlights the potential significance of *Flavonifractor* in the context of diabetic nephropathy. Research on the causal relationship between *Proteobacteria* and nephropathy has been somewhat limited. In a study by Li et al., it was shown that obesity could worsen nephropathy by disrupting the balance of the intestinal microbiota, resulting in an increase in the relative abundance of *Proteobacteria* (18). However, it’s worth noting that their findings contradicted our own results, and this discrepancy might be attributed to the fact that their study was conducted on mice.

Regrettably, no investigation has evidence supporting a genetic correlation between *Sutterella* and nephropathy.

### Strengths and limitations

Our research boasts several notable advantages. First and foremost, our study benefitted from a large sample size and was conducted using a comprehensive GWAS database, ensuring the robustness and reliability of our conclusions. This was particularly advantageous when investigating GM, where previous research had often yielded conflicting results. Second, our MR analyses uncovered previously unknown and meaningful microbial species, such as *Gordonibacter* and two unidentified species. Additionally, we shed light on microbial species that had not been thoroughly investigated in relation to type 2 diabetes complications, such as *Ruminococcaceae* UCG010 and *Dialister*. These discoveries provide valuable directions for future research, offering fresh insights into the complex relationship between GM and type 2 diabetes complications.

There were also some limitations to our study. Firstly, the data used in our study primarily pertained to European populations, which may exhibit distinct microbiota compositions compared to other populations, so our conclusions may not be applicable to other populations. Secondly, the threshold of 5x10^-8^ was considered the gold standard for selecting p-values in MR analyses. However, in our study, setting the GWAS p value at 5 × 10^-8^ during the screening process resulted in an insufficient number of SNPs for the analysis. Therefore, we established the GWAS p value threshold at 1 × 10^-5^. Thirdly, numerous previous studies have suggested a potential link between these three comorbidities (34–36), but in our study, the SNP data for these three complications were sourced directly from the FinnGen database. Each GWAS was conducted independently, and the associations with gut microbiota were analyzed separately.

Moreover, it is crucial to consider the stage, severity, and duration of T2DM in the individuals studied. The gut microbiota can change considerably from the onset and development to the long-term persistence of T2DM and the progression of related complications. Early-stage disorders might exhibit dysbiosis as a causative factor in T2DM and microvascular complications. Conversely, in long-term disorders, alterations in bacterial levels might represent an adaptive response of the microbiota to mitigate systemic dysfunction. These are essential for interpreting our data accurately, considering the significant confounding effects these factors may impose.

Also, the influence of the gut microbiome on pancreatic function and microvascular metabolism is complex and mediated indirectly through an array of bacterial bioactive factors and metabolites. These factors likely act through multiple stages, modulating various aspects of host metabolism, which, in turn, might contribute to the development of retinopathy, neuropathy, or nephropathy. The host’s health and nutritional state also play critical roles in these interactions, potentially influencing the gut microbiome’s impact on disease progression. Individual differences in these host states might explain some of the contradictory findings observed between our study and others. Acknowledging these potential confounding factors provides a more comprehensive understanding of the complex interplay between the gut microbiome and T2DM complications.

The gut microbiota was significantly associated with T2DM microvascular complications through the production of metabolites such as short-chain fatty acids (SCFAs), amino acids, trimethylamine N-oxide (TMAO), bile acids, and indolepropionic acid (37). However, in our study, we focus on the results and discussed the partly metabolites. The remaining gut microbiota will influence the development of microvascular pathologies by releasing which metabolites can be used as a direction for future research. In addition to the well-characterized bacterial constituents of the gut microbiota, other lesser-studied microorganisms such as archaea, mycobionts, and viruses also reside in the gut and may play roles in the pathogenesis of T2DM and its associated microvascular complications, including retinopathy, neuropathy, and nephropathy (38–40). While bacteria dominate the research landscape, these studies suggest that the diverse non-bacterial components of the microbiome could also influence metabolic processes and inflammatory pathways implicated in T2DM. Given the complexity and multifactorial nature of T2DM and its complications, future research should consider these organisms to gain a more comprehensive understanding of the microbial influences at play. Exploring the interactions between these microorganisms and the host may reveal new mechanisms of disease progression and potential therapeutic targets.

## Conclusion

In summary, our MR study provides strong evidence supporting the causal relationship between GM and the development of microvascular complications in T2DM, specifically retinopathy, neuropathy, and nephropathy. These findings underscore the pivotal role that the GM plays in the pathogenesis of these complications. Future research endeavors should focus on delving deeper into the precise mechanisms through which these microbial communities influence the development of microvascular complications. Understanding these mechanisms is essential in developing strategies for the prevention and early diagnosis of these complications.

## Data availability statement

The original contributions presented in the study are included in the article/**Supplementary Material**. Further inquiries can be directed to the corresponding authors.

## Ethics statement

Our study used a publicly available GWAS database that had been approved by the Ethics Committee and no new data were collected, so no additional ethical approval was required.

## Author contributions

JZ: Writing – original draft. ZY: Data curation, Writing – review & editing. SL: Formal analysis, Writing – review & editing. QZ: Supervision, Writing – review & editing. WC: Software, Writing – review & editing. JW: Software, Writing – review & editing. SH: Resources, Writing – review & editing. YL: Resources, Writing – review & editing. SC: Supervision, Writing – review & editing. JX: Funding acquisition, Writing – review & editing.
