# Supplementary figures and images for: Causal relationship between T2DM microvascular complications and gut microbiota: a Mendelian randomization study
Source: Front Endocrinol (Lausanne). 2024 Jun 3;15:1349465. doi: 10.3389/fendo.2024.1349465 (PMC11180823; doi:10.3389/fendo.2024.1349465)

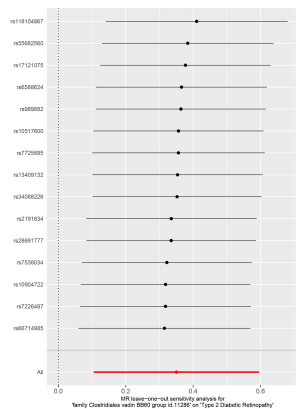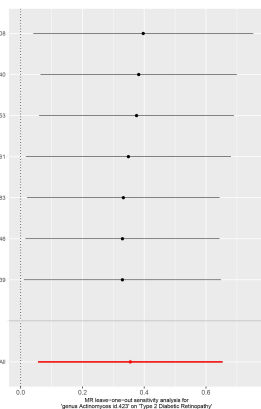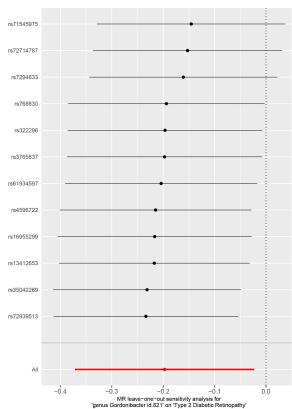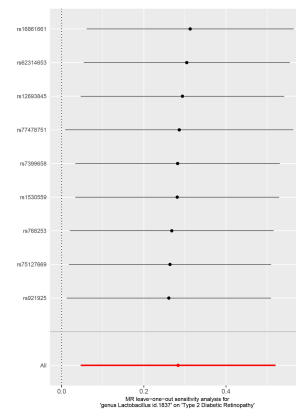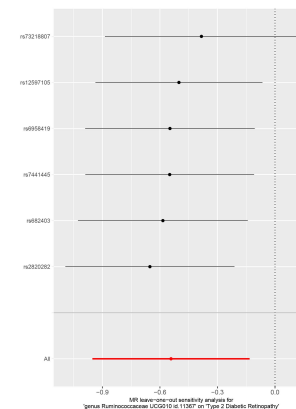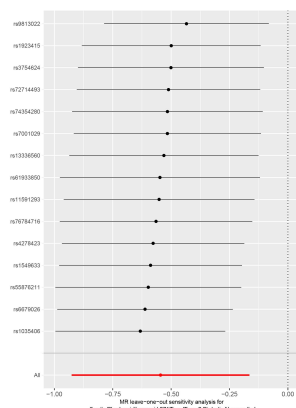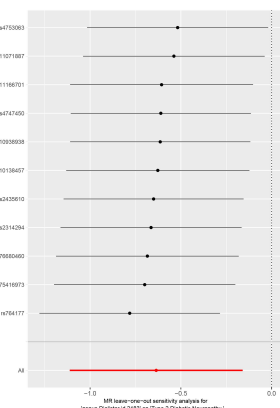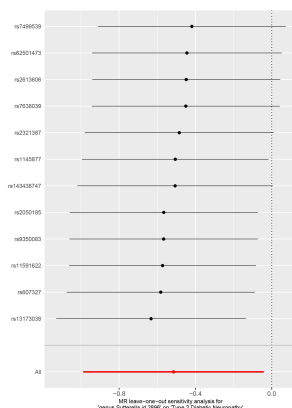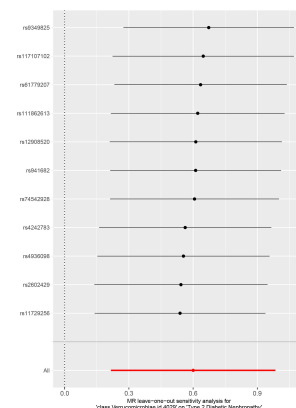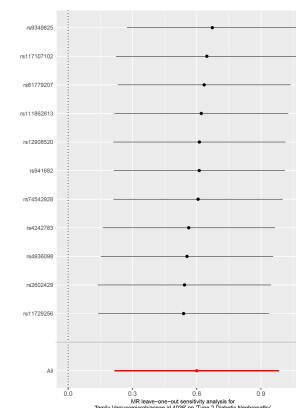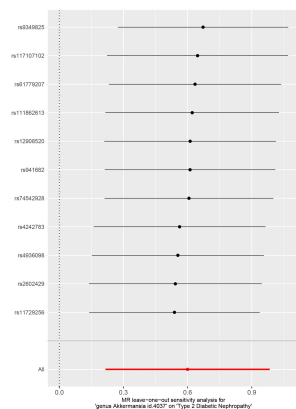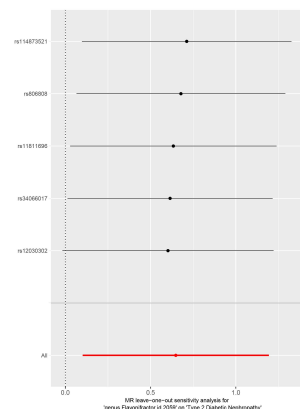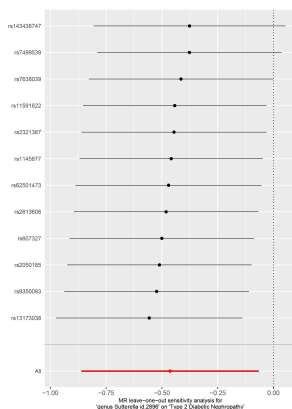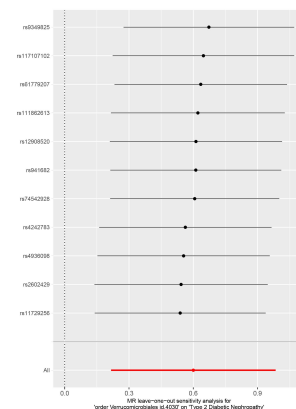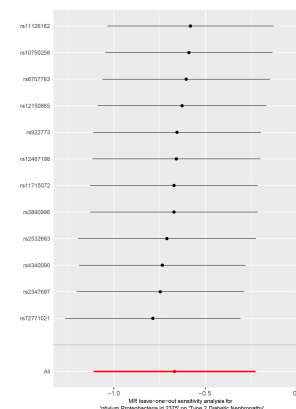

Supplement: Supplementary file 2 [file DataSheet_2.pdf]
